# Fine scale analysis of gene expression in *Drosophila melanogaster *gonads reveals *Programmed cell death 4 *promotes the differentiation of female germline stem cells

**DOI:** 10.1186/1471-213X-12-4

**Published:** 2012-01-17

**Authors:** Amy C Cash, Justen Andrews

**Affiliations:** 1 Department of Biology, Indiana University, Myers Hall, 915 East Third St., Bloomington, IN 47403, USA

## Abstract

**Background:**

Germline stem cells (GSCs) are present in the gonads of Drosophila females and males, and their proper maintenance, as well as their correct differentiation, is essential for fertility and fecundity. The molecular characterization of factors involved in maintenance and differentiation is a major goal both in Drosophila and stem cell research. While genetic studies have identified many of these key factors, the use of genome-wide expression studies holds the potential to greatly increase our knowledge of these pathways.

**Results:**

Here we report a genome-wide expression study that uses laser cutting microdissection to isolate germline stem cells, somatic niche cells, and early differentiating germ cells from female and male gonads. Analysis of this data, in association with two previously published genome-wide GSC data sets, revealed sets of candidate genes as putatively expressed in specific cell populations. Investigation of one of these genes, *CG10990 *the Drosophila ortholog of mammalian *Programmed cell death 4 *(*Pdcd4*), reveals expression in female and male germline stem cells and early differentiating daughter cells. Functional analysis demonstrates that while it is not essential for oogenesis or spermatogenesis, it does function to promote the differentiation of GSCs in females. Furthermore, in females, *Pdcd4 *genetically interacts with the key differentiation gene *bag of marbles *(*bam*) and the stem cell renewal factor *eIF4A*, suggesting a possible pathway for its function in differentiation.

**Conclusions:**

We propose that *Pdcd4 *promotes the differentiation of GSC daughter cells by relieving the eIF4A-mediated inhibition of Bam.

## Background

Stem cells are essential for embryonic development and tissue maintenance and repair. They have the ability to divide to produce cells that can retain stem cell identity (self-renewal) as well as cells that can differentiate into specialized cell types (differentiation). The balance between self-renewal and differentiation is critical. An excess of differentiation can lead to stem cell depletion and tissue senescence, whereas an excess of self-renewal, and/or a deficiency of differentiation, can lead to an accumulation of undifferentiated proliferative cells. The Drosophila germline is a well established model for the study of adult stem cells and the control of self-renewal and differentiation [1].

Many stem cells depend on a specialized microenvironment, the stem cell niche. Drosophila germline stem cells are sexually dimorphic and exist in sexually dimorphic niches. Each ovary is composed of approximately 16 tubes called ovarioles. At the anterior of the ovariole, the germarium houses the stem cell niche, germline and somatic stem cells, and the first stage of egg chamber development. Somatic cells, including terminal filament cells, cap cells, and escort stem cells, form the stem cell niche and provide a cellular environment that is essential for the maintenance of the 2-3 germline stem cells (GSCs) that occupy the niche (reviewed in [2,3]). The maintenance of GSCs requires adherens junctions with niche cells and short-range BMP signaling from the niche. This signaling acts to repress expression of the key differentiation gene *bag of marbles *(*bam*) in the stem cells. Typically the GSCs divide asymmetrically. The daughter cell that maintains contact with the cap cells retains GSC identity. The daughter cell that is displaced posteriorly loses cell contact and signaling from the niche and then initiates differentiation with the up-regulation of *bam *expression. Cells that are removed from the stem cell position but have not yet accumulated high levels of Bam are referred to as pre-cystoblasts (pre-CBs), and once they have accumulated high levels of Bam, they are referred to as cystoblasts (CBs) [4,5]. The CBs then undergo four incomplete mitotic divisions and form a 16-cell cyst. This cyst gets encapsulated by somatic follicle cells and buds off of the germarium as a stage 1 egg chamber, which then undergoes extensive growth and differentiation to eventually produce an egg. In males, the stem cell niche and the germline and somatic stem cells are located at the closed anterior apex of each testis. The stem cell niche that supports self renewal of GSCs and somatic stem cells is formed by approximately 20 somatic "hub" cells [6]. As in females, the GSCs are in physical contact with and receive short-range signals from the niche cells. In males, however, the predominant signals come from the JAK/STAT signaling pathway. Male GSCs also divide asymmetrically, such that the daughter cell maintaining contact with the hub retains stem cell identity. The daughter cell that is displaced away from the hub, called the gonialblast, loses maintenance signals from the niche, and is encapsulated by somatic cyst cells. During early differentiation, each gonialblast undergoes four rounds of incomplete mitotic division (during which the cells are called spermatogonia) to form a cyst of 16 primary spermatocytes. The primary spermatocytes then undergo extensive cell growth and the transcriptional activation of a large repertoire of testis-specific genes. Primary spermatocytes eventually undergo meiosis, forming spermatids and ultimately sperm. In both female and male gonads, the coordinated action of all the various cell types is essential for proper gametogenesis.

Forward genetic screens have identified many key regulators of both stem cell maintenance and the early differentiation programs of female and male germ cells in Drosophila. These have identified genes that function in specific cell types such as GSCs, gonadal somatic cells, or differentiating germ cells in either females, males, or both sexes. While the genetic approach has been highly fruitful, profiling genome-wide expression patterns can provide a valuable complement. Spatial and temporal expression patterns have been used to infer the likely site(s) of action of the corresponding gene products [7-14]. We wished to identify additional candidate genes based on expression patterns in specific gonadal cell populations. In metazoans, the ability to profile genome-wide expression patterns in specific cell types is limited by the ability to isolate or purify RNA from specific populations of cells. This can be achieved by physically collecting specific cells or tissues either by cell culture [15,16], dissection [7,12,17,18], laser cutting/capture [19,20], cell sorting [9,21,22], or by purification of tagged RNA [22,23]. Genetic manipulations may also be used to enrich for specific cell types, and may also be used in concert with physical collection approaches [14,24]. Alternatively, the computational integration of expression data across multiple tissue samples and/or conditions can be used to identify cliques of genes that are co-expressed in particular cell populations or conditions [17,25-27]. Here we have used a combination of approaches - expression profiling of isolated wild-type cell populations and data integration - to identify genes expressed in specific germline and gonadal cell populations.

We used laser cutting and RNA amplification as a means of profiling gene expression in wild-type female germaria and male apex of the testis (the anterior-most region of the testis that includes somatic cells, germline stem cells, mitotically dividing germ cells, and some primary spermatocytes). As expected, there was a high degree of sex-biased gene expression. We analyzed our expression data with respect to previous genome-wide data sets in order to identify sets of genes co-expressed in specific cell populations, including GSCs, early differentiating germ cells, and somatic cells. We investigated the function of one of these genes, *CG10990 *the Drosophila ortholog of the eukaryotic tumor suppressor gene *Pdcd4*. This revealed that while *Pdcd4 *is not essential for oogenesis or spermatogenesis, in females, it interacts genetically with *bam *and *eIF4A*, and its function is required for the efficient differentiation of GSC daughter cells.

## Methods

### Fly Stocks and Genetics

Flies were raised on standard corn meal agar medium at 25°C. Wild-type flies were Oregon-R. The following mutant stocks were used: *CG10990^G93 ^*(GFP protein trap line G00093 [28]), *Tm1^ZCL0722 ^*(GFP protein trap line ZCL0722 [28]), *CG1600^CB03410 ^*(GFP protein trap CB03410 [8]), *nrv2^ZCL2903 ^*(GFP protein trap line ZCL2903 [28]), *GlcAT-S^CA07168 ^*(GFP protein trap line CA07168 [8]), *Df(1)ED7217/FM7 *(Bloomington Drosophila Stock Center, 8952*), bam^Δ86^/TM3 *(Bloomington Drosophila Stock Center, 5427), *eIF4A^1013 ^*(Bloomington Drosophila Stock Center, 8647), *bam-GFP *(provided by Michael Buszczak, University of Texas Southwestern Medical Center). The new allele *Pdcd4^1 ^*was generated using the FLP-FRT deletion strategy [29,30] using the insertions *PBac{WH}CG10990^f06108 ^*and *P{XP}CG10990^d04016 ^*(Exelixis Collection, Harvard Medical School), and the *P{ry^+t7.2 ^= hsFLP}86E *transgene (Bloomington Drosophila Stock Center, 279) as the source of flipase. The presence of the new recombinant hybrid element was detected by PCR using the following hybrid element-specific primers: forward: AATGATTCGCAGTGGAAGGCT, reverse: GACGCATGATTATCTTTTACGTGAC. Presence of the recombinant chromosome containing the hybrid element and lacking the *Pdcd4 *(*CG10990*) sequence was confirmed first by PCR amplification using gene specific primers (forward: GGCACAACAAGTGCGAAAGAAGGA, reverse: CGCTTGCGTCCGCTGCAAATATAA) and then sequencing the product.

### Laser Cutting Microdissection

Ovaries and testes were dissected from 24-48 hour old wild-type flies in 1× phosphate buffered solution (PBS, Ambion), and ovaries were separated into individual ovarioles. Tissue was fixed in 95% ethanol for 5 minutes. Individual ovarioles and testes were transferred in 95% ethanol to metal-framed PEN membrane slides (Molecular Devices), and the ethanol was allowed to evaporate at room temperature for 10 minutes. Laser cutting microdissection was performed using the Arcturus Veritas Microdissection System (Molecular Devices). Germaria were visualized by light microscopy and cut with the UV laser, using a power setting between 3.5 and 5.5. Testes were visualized by light microscopy and the apical-most 50 μm of testis tissue was cut with the UV laser, using a power setting between 6 and 7. Tissue samples were attached to CapSure Macro Caps (Molecular Devices) using the IR laser, and only one piece of tissue (either a single germarium or single testis apex) was attached to a single cap. The caps were placed into thin-wall microcentrifuge tubes (Applied Biosystems) containing 50 μl of extraction buffer (PicoPure RNA Isolation Kit, Molecular Devices) and RNA was extracted immediately. All steps from tissue fixation to the beginning of total RNA extraction were completed within 2 hours.

### RNA Extraction and Amplification

Total RNA from laser cut samples was extracted using the Pico Pure RNA Isolation Kit (Molecular Devices). The extraction buffers of 4 individual pieces of tissue were pooled together before total RNA extraction, such that 1 biological sample contained either 4 germaria or 4 testes apex. In total, 4 independent biological samples were prepared for each tissue type. After sample pooling, total RNA extraction was performed according to standard Pico Pure RNA Isolation kit protocol. Total RNA was eluted in 15 μl EB Buffer (Molecular Devices). The quality of RNA was analyzed using a Pico Chip on the Agilent 2100 BioAnalyzer. Total RNA was stored at -80°C until amplification.

RNA amplification was performed for samples generated by laser cutting. Total RNA was incubated with 100 ng T7-oligo(dT) primer (Ambion) at 65°C for 10 min and then cooled on ice. First strand DNA synthesis was performed by adding 2 μl 5× first strand buffer (Invitrogen), 1 μl 0.1 M DTT, 1 μl 10 mM dNTPs, 0.3 μl T4gp32 (USB, 13.4 μg/μl), 0.7 ul RNase Inhibitor (Ambion, 10 U/μl), and 1 μl Superscript II (Invitrogen, 200 U/μl), followed by incubation at 42°C for 2 hr, 65°C for 10 min, and then cooled on ice. Second strand DNA synthesis occurred by the addition of 64 μl of second-stand master mix (45 μl water, 15 μl 5× second strand buffer, 1.5 μl 10 mM dNTPs, 2 μl DNA polymerase (Invitrogen, 10 U/μl), and 0.5 μl RNaseH (Invitrogen, 2 U/μl)), followed by incubation at 16°C for 2 hr. cDNA clean-up was performed with MiniElute Reaction Cleanup columns (Qiagen) and eluted in 10 μl of elution buffer. The volume was increased to 16 μl by addition of 7 μl of RNase-free water. *In vitro *transcription (IVT) was performed using the MEGAscript kit (Ambion) with a 4 hour incubation at 37°C. Amplified RNA (aRNA) was purified with the RNeasy kit (Qiagen) and eluted in 100 μl RNase-free water. After adjusting the aRNA volume to 4 μl using a Speed Vac, 3 μg random primers (Invitrogen) were added and incubated at 65°C for 10 min and cooled on ice. 6 μl of first-strand mix was then added and reactions were incubated at 42°C for 2 hours. Second round second strand synthesis, cDNA purification, IVT, and aRNA purification were all carried out as in the first round, with the following exception: the last wash of the aRNA purification was performed with 80% ethanol as opposed to Qiagen buffer RPE. The final amplified antisense RNA product was quantified using a Nanodrop (Thermo Scientific).

### Microarrays

Four microarray replicates comparing WT germaria to WT testes apex were performed, with two dye flips. Amplified RNA (aRNA) was directly labeled with Cy3 or Cy5 using the Kreatech ULS aRNA Fluorescent Labeling Kit (Biomicrosystems). We labeled 2 μg of each aRNA sample with Kreatech Cy3 or Cy 5-ULS (Biomicrosystems). For microarray hybridization, samples were balanced for labeling efficiency, such that 50 pmol of dye for each sample were hybridized. Hybridization conditions and post-hybridization washes were as described in Kijimoto et al. 2009 [31] at a temperature of 42°C. Rinsed microarray slides were dried by centrifugation at 500 rcf for 4 min. Samples were hybridized to Drosophila Genomics Resource Center (DGRC) oligonucleotide microarrays https://dgrc.cgb.indiana.edu/microarrays/platforms.html.

Microarrays were scanned using a GenePix Scanner 4200 (Molecular Devices) with GenePix Pro 5.0 software. Data was normalized using OLIN in Bioconductor https://dgrc.cgb.indiana.edu/microarrays/support/bha.html. Statistical analyses were performed using the limma modified t-test [32] with a false discovery rate correction. An FDR cutoff of < 1% was used for statistical significance. The expression difference between 2 samples is given as a log_2 _ratio between the two signal intensities (M value). Microarray data is available at GEO, accession number GSE22686. Gene Ontology (GO) analysis was performed using the GO Fat option in DAVID [33,34]. Biological Process GO terms were identified as over-represented if they had an FDR-corrected p-value < 0.01.

Affymetrix microarray data from Terry et al. [14] and Kai et al. [9] were processed as follows. Raw data files were collected from the corresponding authors. Presence/absence calls were processed through standard Affymetrix data methods (Affymetrix, 2004. Expression analysis technical manual. http://www.affymetrix.com/support/technical/manual/expression_manual.affx). Data was normalized using GCRMA [35] and analyzed using the limma package in Bioconductor. Affymetrix data from Kai et al. was analyzed such that *dpp*-expanded and *bam*-mutant data were pooled and analyzed together to form a female GSC data set and that was then compared to the Kc cell data. Data from Terry et al. was analyzed such that *Os^+^bgcn^- ^*data were directly compared to the *bgcn^- ^*data, resulting in a set of male GSC, gonialblast, and somatic stem cell enriched genes. All analyses included a false discovery rate correction and an FDR cutoff of < 1% for statistical significance.

### *In situ *Hybridization

DNA templates for the preparation of *in situ *probes were prepared by PCR amplification using the following primers:

*CG7777*: forward ATTCTCATCGATCAGGACTATTAC, reverse GGTACTTCTCGGATGCCTCGTTA

*CG4404*: forward GAAGTATCTTCAGTTTCTCGTTCC, reverse GTGAAAACACTTAGAGCTGACGTA

*CG9975*: forward CGTTATTTAGTACAGGCAATTCAG, reverse GCACTAAGTCCACTCATTTTACAT

*Pp1-13C*: forward AAGCGAGAATTTGTCCTACATT, reverse GCTCTCCAAATTGAGAACCT

*CG9925*: forward TTGAGTAAGGATAGTGGATTGTTC, reverse CAGAAGTAGAGACTGTGGAGACAC

*CG10990 (Pdcd4*): forward TAACGAGGAACGTATATATCGAAG, reverse GTAGTTAGGATCATTCTCGTCCTC

PCR products were cloned into pCR II-TOPO plasmid (Invitrogen) using the TOPO TA Cloning kit (Invitrogen). Digoxigenin-labeled RNA was prepared using the Sp6/T7 DIG RNA labeling kit (Roche). *In situ *hybridization was performed as described in [36] using an alkaline phosphatase-conjugated anti-digoxigenin antibody (Roche). RNA *in situ *staining was examined using bright field and differential interference contrast (DIC) microscopy.

### Immunohistochemistry

Ovaries and testes were dissected from 5 day old mated females and males in PBS and fixed in a 4% paraformaldehyde solution for 45 minutes. They were rinsed in PBT (0.1% Tween-20 in PBS), placed in blocking solution (10% normal goat serum) for 10 minutes and incubated overnight with primary antibodies. Samples were then rinsed in PBT for 10 minutes, incubated with secondary antibodies for 3 hours, and rinsed again in PBT for 10 minutes. Primary antibodies included: polyclonal rabbit anti-GFP (1:200, abcam 290), monoclonal rat anti-Vasa (1:100; Developmental Studies Hybridoma Bank), monoclonal mouse anti-FasIII 7G10 (1:50; Developmental Studies Hybridoma Bank), monoclonal mouse anti-Hts antibody 1B1 (1:20; Developmental Studies Hybridoma Bank), and polyclonal rabbit anti-pSmad1/5/8 (1:300; provided by E. Laufer, Columbia University). Secondary antibodies included: rhodamine-conjugated goat anti-rat (subtracted to prevent cross-reactivity), fluorescein-conjugated goat anti-mouse (subtracted to prevent cross-reactivity), rhodamine-conjugated goat anti-mouse, fluorescein-conjugated goat anti-rabbit, and rhodamine-conjugated goat anti-rabbit (all at 1:100, Jackson ImmunoResearch). Microscopy was performed on a Leica SP5 scanning confocal microscope.

## Results

### Gene expression patterns in GSCs and early gametogenesis

With the aim of identifying new candidate genes with putative functions in stem cell maintenance and the early differentiation programs of female and male germ cells, we sought to identify genes whose expression is enriched in specific cell populations in adult gonadal tissue containing GSCs, early differentiating germ cells, and somatic cells. Previous genome-wide gene expression studies in flies have examined either whole gonads [7,11], hand dissected regions of gonads [37], or stem cells enriched by cell sorting and/or genetic manipulation [9,14]. The use of whole organs precludes cell specific information, and the use of genetic or physical manipulation risks disturbing normal expression patterns. We sought to circumvent these limitations by pursuing a two-pronged approach. First, we examined gene expression in the early stages of gametogenesis in wild-type females and males through fine scale laser microdissection. This had the advantage of isolating tissues of interest - germaria and apex of the testis - without genetic manipulation and minimal physical manipulation (prior to tissue fixation). Second, we integrated this new wild-type data with previously published complimentary genome-wide data sets. This had the advantage of allowing us to identify genes with inferred expression in particular cell types in early gametogenesis.

In the first approach, we examined gene expression in the early stages of gametogenesis in wild-type females and males. Whole germaria and apex of the testes were captured by laser cutting microdissection. RNAs were isolated from these tissues and then amplified, labeled, and hybridized to microarrays. The concordance between four independent biological replicates of each tissue sample revealed that the data were highly reproducible. For the replicate germarium and apex of the testis samples, the average R value was 0.90 (range: 0.86-0.94) and 0.92 (range: 0.88-0.96), respectively.

**Figure 1:**
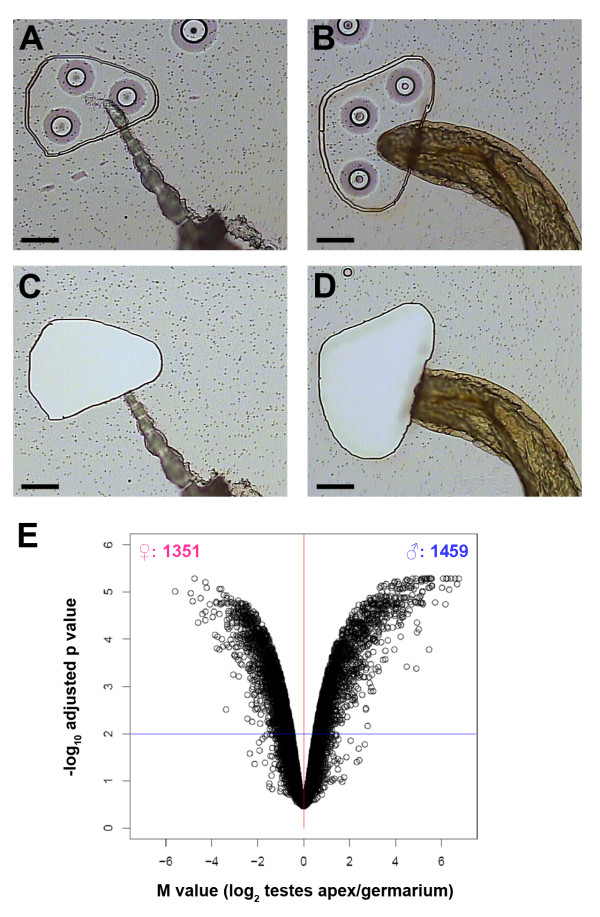
**Gene expression in the germarium and apex of the testis**. **(A-D) **Laser cutting microdissection. Photomicrographs show fixed and dehydrated ovariole (A, C) and testis (B, D), before (A, B) and after (C, D) the tissue was captured. Scale bars are 50 μm. The captured tissue was used for RNA isolation and subsequent expression profiling. **(E) **Differential gene expression in the germarium compared to the testis apex. Volcano plot shows M value (log_2_(testis apex/germarium) on the X-axis and statistical significance (-log_10 _adjusted p-values) on the Y-axis.

Before examining sex-biased gene expression, we examined markers known to be expressed in a stage-specific manner during spermatogenesis in order to assess the cell types included in the testis samples. Since the germarium is a morphologically distinct structure, we were able to readily dissect whole germaria, although we could not be certain that the terminal filament was included (Figure 1). Thus, we were confident that the female samples included germarial somatic cells, GSCs, early differentiating germ cells and newly formed egg chambers. On the other hand, the testis lacks distinct morphological landmarks; the hub and the gonial proliferation center (GPC, which includes GSCs and spermatogonia) are located at the apex of the testis and primary spermatocytes, spermatocytes and spermatids are progressively displaced distally. We dissected the apical-most 50 μm (Figure 1) which would be expected to encompass the hub, the gonial proliferation center, and possibly some primary spermatocytes, but to exclude meiotic cells or spermatids. In order to assess the degree to which our testis sample included primary spermatocytes, we examined microarray intensity values of two groups of genes with known patterns of expression during spermatogenesis. The first group are expressed throughout the GPC (Table One in Terry et al. [14]) and the second group are expressed starting in primary spermatocytes (testis-specific TAFs in [38,39]). GPC-expressed genes included *fng*, *kek1*, *neur*, *aop*, *apt*, and *Imp *and had an average intensity value of 490 (range 94-1332). Genes expressed in primary spermatocytes included *can*, *mia*, *nht*, *sa*, and *Taf12L *and had an average intensity value of 4646 (range 486-18,742). These data indicate that the male tissue did contain primary spermatocytes in addition to the cells located within the GPC.

We then examined sex-biased gene expression between the samples. In the direct microarray comparison between wild-type tissue from female germaria and male apex of testes, 2,810 genes were identified as significant using a false discovery rate adjusted p-value of < 0.01. Of these, 1,351 were preferentially expressed in the germaria and 1,459 were preferentially expressed in the apex of the testes (Figure 1, Additional File 1).

As expected, genes preferentially expressed in the germarium included many genes with known function in the female germline. Analysis of over-represented Gene Ontology (GO) Biological Process terms (complete list of GO terms in Additional File 2) from germarium-biased genes revealed numerous categories specific to ovary tissue, including: oogenesis (FDR-corrected p-value < 4.49 × 10^-9^), female gamete generation (corrected p-value < 5.58 × 10^-9^), ovarian follicle cell development (corrected p-value < 2.61 × 10^-6^), and germarium-derived egg chamber formation (corrected p-value < 4.37 × 10^-3^). Also included were embryonic morphogenesis (corrected p-value < 1.10 × 10^-5^), embryonic axis specification (corrected p-value < 3.45 × 10^-3^), embryonic pattern specification (corrected p-value < 4.56 × 10^-3^), anterior/posterior pattern formation (correct p-value < 3.13 × 10^-4^), and dorsal/ventral pattern formation (corrected p-value < 7.27 × 10^-3^), all of which indicate that the genes for oocyte patterning and embryonic development are turned on and highly differentially expressed at the earliest stage of oogenesis. No GO terms associated with male gametogenesis were found in our list derived from germarium-biased genes. Genes with high differential expression in the germarium include genes known to be involved in female germline sex determination such as *ovo *and *ovarian tumor *[40,41]. Genes involved in oocyte axis determination, either through their own localization (*bicoid*, *oskar*, *gruken*, and *orb*) [42-44] or by aiding in mRNA localization (*egalitarian*, *Bicaudal D*, *swallow*, *Lis-1*) [43,44], are highly differentially expressed. Genes functioning in female fertility and sterility, such as *alpha-tubulin67c *[45], *deadhead *[46], *fs(1)M3 *and *fs(1)N *are also differentially expressed in the germarium, as are all three genes of the PNG kinase complex (*pan gu*, *plutonium*, and *giant nuclei*), which are maternally required for early embryonic cell division [47-49]. The genes preferentially expressed in the germarium also included many genes not previously known to function in the female gonad. First, these genes included 568 previously unstudied genes ("CG genes"). Second, 24.6% of the germarium-biased genes did not show ovary-biased gene expression in previously published microarray data from whole adult gonads [18] and represent genes with female-biased expression detectable only in early gametogenesis. These genes are candidates for future studies of early oogenesis.

In the list of differentially expressed genes in the apex of the testis, we found 10 over-represented Biological Process GO categories with a FDR-corrected p-value < 0.01 (Additional File 2). These include spermatogenesis (corrected p-value < 8.45 × 10^-4^) and male gamete generation (corrected p-value < 8.45 × 10^-4^). The other significant GO categories related to various catabolic processes. We did not find any over-represented GO categories associated with microtubules, microtubule-based movement, or cytoskeleton, which could all be associated with fully developed sperm and sperm tails. This indicates that our area of laser cut tissue does not contain developing spermatids. Genes with differential expression in the apex of the testis include genes known to be testis-specific TBP-associated factors (TAFs), such as *Taf12L (*also known as *rye*), *no hitter *(*nht*), and *spermatocyte arrest *(*sa*) [38]. Genes with male-specific germline functions, such as *tomboy20 *[50], *beta-tub85D *[51], and *don juan *[52], are also differentially expressed. In addition, several genes identified as male germline specific transcripts, *Mst87F *[53], *Mst98Ca *and *Mst98Cb *[54], and *Mst84Da*, *Mst84Db *and *Mst84Dc *[55], have some of the highest differential expression values in the experiment. The presence of these significant genes confirms that we are detecting genes with sex-specific or sex-biased functions in early male germline cells. The genes preferentially expressed in the apex of the testis also included many genes not previously known to function in the male gonad. First, these genes included 915 previously unstudied CG genes. Second, 37% of the apex of the testis-biased genes did not show whole testis-biased gene expression in previously published microarray data from whole adult gonads [18] and represent genes with male-biased expression detectable early in gametogenesis. These genes are candidates for future studies of early spermatogenesis.

**Figure 2:**
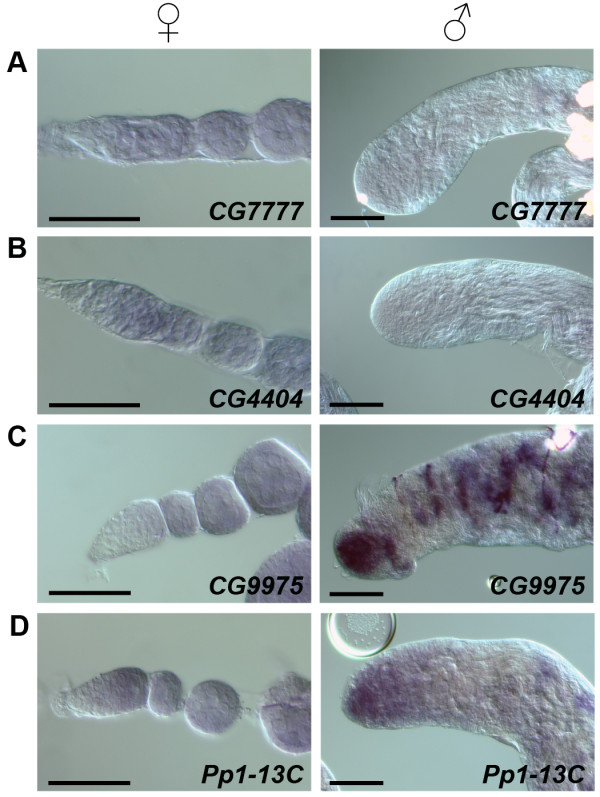
**mRNA expression patterns of sex-biased candidate genes in ovaries and testes**. **(A-D) **Light micrographs showing *in situ *hybridization to *CG7777 *(A), *CG4404 *(B), *CG9975 *(C) and *Pp1-13C *(D) in ovary (left panel) and testis (right panel) tissue. Scale bars are 50 μm.

The genes identified here potentially have sex-specific functions in the earliest stages of gametogenesis. We tested the expression pattern of four highly sex-biased genes, including three previously unstudied CG genes. Both *CG7777*, inferred to be a member of the aquaporin family of water channels based on its sequence similarity to *Drip *[56], and *CG4404*, inferred to have DNA binding activity, were significantly germarium-biased. RNA *in situ *hybridization of *CG7777 *revealed expression throughout the germarium and early egg chambers but no expression in the apex of the testis (Figure 2A). Expression of *CG4404 *was very similar with expression in the germarium and early egg chambers but no expression in the testis (Figure 2B). We also tested two of the apex of the testis-biased genes. *CG9975 *is completely uncharacterized and *Pp1-13C *encodes a protein phosphatase 1 catalytic subunit. *CG9975 *is expressed at a very high level in the apex of the testis and also in later stages of spermatogenesis. However, no expression was seen in the first two-thirds of the germarium and only low levels of expression were observed in the last third of the germarium (Figure 2C). The expression of *Pp1-13C *is highest in the apex of the testis and gradually decreases. Within the germarium, there is weak to no expression in the anterior tip and a low level of expression in the rest of the germarium (Figure 2D). These *in situ *results verify our microarray data and provide us with several interesting candidate genes for sex-biased early germline function.

The germarium and apex of the testis contain many cell types including several somatic cells, GSCs, and differentiating germline cells. Genes showing expression in these structures could be expressed in one or more of the constituent cell types. In the second approach of our study, we analyzed our data with respect to data from previous studies of gene expression in stem cells and their daughter cells in order to begin to analyze the expression patterns in different cell types. Kai et al. [9] used flies over-expressing *dpp *or mutant for *bam *to generate ovaries with expanded germline stem cell populations and then used fluorescence-activated cell sorting (FACS) to isolate the stem cells expressing vasa-GFP. Gene expression levels in these cells were compared to those in Kc cells. Terry et al. [14] used whole genome microarrays on *bgcn*-mutant testes and *bgcn*-mutant testes over-expressing the Jak/Stat activator *Os *to study the expression of testes enriched in germline stem cells, their daughter cells, and somatic stem cells. Examining expression patterns across these experiments can be used to infer whether genes are expressed in wild-type germline stem cells, daughter cells, or other cell types in the germarium and/or testis. Therefore we combined our data with these two data sets and performed several filtering analyses to highlight genes expressed in specific cell populations. Our first filtering analysis sought to identify genes whose expression is enriched in wild-type GSCs and/or daughter cells in both female and male germline stem cell niches. Our criteria for inclusion in this group required that genes be enriched and significant in the GSC comparison from Kai et al. (*dpp*-expanded and *bam*-mutant germ cells vs. Kc cells) and the stem cell/gonialblast comparison from Terry et al. (*Os^+^bgcn^- ^*testes vs. *bgcn^- ^*testes). Genes were also required to have a baseline level of expression in our data set, with an intensity value greater than 64 (log_2_(intensity) > 6) for both the germarium and apex of the testis. It should be noted that while meeting these criteria requires that genes are preferentially expressed in GSCs and/or daughter cells, it does not require that they are expressed exclusively in these cells. A total of 391 genes met these criteria. We then rank ordered these genes by their log_2_(ratio) values (M value) in Kai et al. and Terry et al. and summed those rank orders for the final ranked list (Additional File 3). Many genes at the top of this rank ordered list, including *piwi*, *ovo*, *aub*, *vasa*, and *neur*, are known to be involved in both female and male germline tissue, including germline stem cells and niches. For example, the first rank ordered gene is *piwi*, which is expressed in the germarium and apex of testis and is an essential factor in germline stem cell maintenance in both females and males [57-59]. The gene *ovo *is expressed in the germline stem cell niches of both females and males although it doesn't appear to play a role in the male germline [60,61]. The gene *aub*, a member of the Argonaute family, is involved in silencing transposons in the germline and is expressed throughout the germarium and apex of testis [62-64]. The gene *vasa *is a marker of primordial germ cells and germline stem cells [65,66], and the *neur *gene has been shown to be expressed in male GSCs and early gonial cells [14] and female somatic polar follicle cells and germarium cells [67]. The genes in this list also included many genes not previously known to function in both female and male GSCs, including 147 previously unstudied CG genes. The inclusion of the known genes in our filtering analysis supports the inference that additional genes identified in this gene set may also function in germline stem cells and/or daughter cells of females and males.

**Figure 3:**
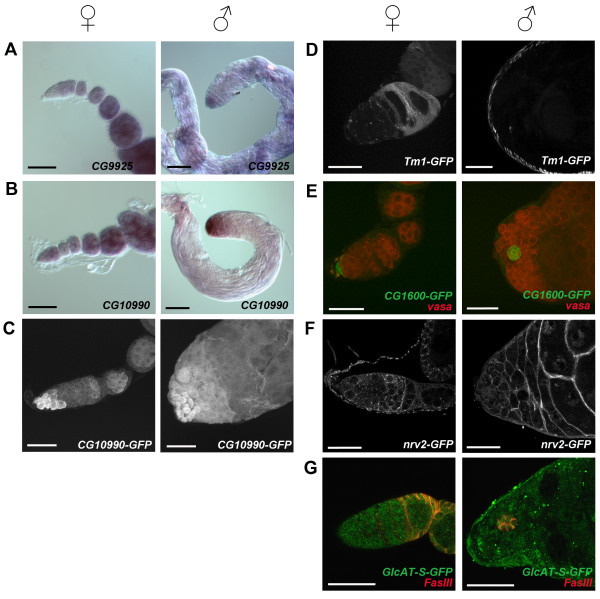
**Expression patterns of candidate genes in ovaries and testes**. **(A-B) **Light micrographs showing *in situ *hybridization to *CG9925 *(A) and *CG10990 *(B) in ovary (left panel) and testis (right panel) tissue. Scale bars are 50 μm. **(C-G) **Immunofluorescence images of GFP protein traps [8,28] reporting expression of CG10990 (C), Tropomyosin I (Tm1; D), CG1600 (E), nervana 2 (nrv2; F) and GlcAT-S (G) in ovary (left panel) and testis (right panel) tissue. Immunostained proteins are listed at the bottom right of each panel. Immunofluorescence images are either confocal projections (C-E) or single Z-stack images (F-G). Scale bars are 25 μm.

We examined the expression of two previously uncharacterized genes within this gene set, *CG9925 *and *CG10990*, in whole adult gonads by RNA *in situ *hybridization. We also examined the protein expression of *CG10990 *through the use of a protein trap reporter [28]. In females, *CG9925 *mRNA is expressed in the germarium and throughout the developing egg chambers (Figure 3A). In males, *CG9925 *is expressed within the testis, with high levels at the apex in the region containing the hub, GSCs, and early mitotically dividing germ cells (Figure 3A). RNA *in situ *hybridization revealed that *CG10990 *mRNA is expressed in germ cells in both females and males (Figure 3B). In females, the RNA is expressed at all stages of oogenesis. Expression in males is limited to the apex of the testis with the highest levels encompassing the gonial proliferation center (GPC). The CG10990-GFP protein trap line showed strong expression of the GFP fusion protein in the anterior-most region of the ovaries and testes (Figure 3C). In females, protein expression was highest in cells at the anterior tip of the germarium. The signal then decreased dramatically in the middle region of the germarium and returned at a lower level in germ cell cysts in the posterior of the germarium. This lower level of expression was seen in all subsequent stages of egg chamber development. In males, protein expression was highest in the germline stem cells and then decreased in the GPC. The expression patterns of both of these genes suggest they may be functioning in the earliest stages of adult gametogenesis.

Examining expression patterns across our data and the data from Kai et al. [9] can also be used to identify genes that are enriched in the germarium but not female GSCs. Our data contains all of the wild-type cells within the germarium, including GSCs, differentiating germ cells, and somatic cells, but the data from Kai et al. only contains GSC-like germ cells. By filtering the data from these two experiments, we can identify genes with expression putatively enriched in female somatic cells and differentiating germ cells. To identify these genes, we filtered our data and the data from Kai at al. to include genes enriched and significant in the germarium and exclude genes enriched and significant in the GSC comparison from Kai et al. This analysis identified 554 genes, and the genes were rank ordered by their M value in the germarium vs. apex of the testis comparison (Additional File 4). There were 15 GO terms over-represented in this gene set. They include: post-embryonic morphogenesis, mesoderm development, imaginal disc morphogenesis, appendage development, and wing disc development. These terms were identified, in part, because of the inclusion of a large number of genes associated with signaling pathways, such as EGFR signaling (*spitz *(*spi*) and *rhomboid *(*rho*)), Dpp signaling (*Daughters against dpp *(*Dad*) and *thickveins *(*tkv*)), Notch signaling (*Delta *(*Dl*)), Wingless signaling (*frizzled *(*fz*)), and Hedgehog signaling (*hedgehog *(*hh*) and *cubitus interruptus *(*ci*)). Hedgehog signaling plays a very important role in the germarium as *hh *is expressed in terminal filament and cap cells and directly stimulates the proliferation of ovarian somatic cells, and *ci *is a transcription factor that acts downstream of Hedgehog signaling [68-70]. Notch signaling is required for the formation and maintenance of the niche, and in the adult germarium, *Dl *is expressed predominantly in niche cells [71,72]. In addition, EGFR signaling is required for patterning the eggshell, and in the adult germarium, the ligand *spi *is expressed in follicle cells starting in region 2 [73,74]. However, it should be noted that while the Dpp signaling components *Dad *and *tkv *were identified in this gene set and thus classified as being putatively enriched in somatic cells or differentiating germline cells, *Dad *is expressed predominantly in GSCs in the adult germarium [75,76], and *tkv *is required in GSCs for GSC maintenance [77]. They were placed in this category because they were not found to be significantly enriched in female GSCs in the Kai et al. data. Overall this gene list, including 198 previously unstudied CG genes, identifies candidate genes that may function in the germarium in somatic cells or differentiating germ cells.

We examined the expression of two additional genes within this gene set to determine if they were expressed in germarial somatic and/or differentiating germ cells. The gene *Tropomyosin 1 *(*Tm1*) is a component of the actin cytoskeleton and is required for *osk *mRNA localization to the posterior pole of the oocyte [78,79]. We examined the expression of *Tm1 *within the germarium using a GFP protein trap reporter [28] and found that it is expressed in follicle cells in the second half of the germarium (Figure 3D). In males, we found *Tm1 *to be expressed in the epithelium surrounding the testis (Figure 3D). Our gene list also included *CG1600*, which has been identified as expressed in cap cells through the use of a GFP protein trap reporter [8]. To confirm this and examine its expression in testis, we performed antibody staining with Vasa and GFP (Figure 3E). Within the germarium, *CG1600 *is clearly expressed in cap cells and not germ cells (shown in red with Vasa staining). Interestingly, it is also expressed exclusively in hub cells in the testis with no expression in germ cells. The expression patterns of both of these genes support their inclusion in this set of genes and suggest other genes in this list may be expressed in somatic cells or later differentiating germ cells within the germarium.

We used a similar filtering analysis with our data and the data from Terry et al. [14] to identify genes whose expression is enriched in the apex of the testis but not in male GSCs, gonialblasts, or somatic stem cells (the cell types enriched in *bgcn*-mutant testes over-expressing *Os *compared to *bgcn*-mutant testes). We filtered our data and the data from Terry et al. to include genes enriched and significant in the apex of the testis from wild-type flies and exclude genes enriched and significant in male stem cells and gonialblasts from the analysis of Terry et al. This analysis identified 630 genes. Genes significantly enriched in the apex of the testis were rank ordered by their absolute M value. Genes were also rank ordered by their M value in the *bgcn*-mutant testes over-expressing *Os *compared to *bgcn*-mutant testes, with *bgcn*-mutant enriched genes at the top (this should highlight those genes enriched in later differentiating stages). These rank orders were summed to determine the final ranked list (Additional File 5). This analysis is expected to identify genes expressed in somatic cells and/or later differentiating germ cells within the apex of the testis. There were no over-represented GO terms in this gene set. Several of the genes included in this list were *Taf12L*, *r-cup*, and *f-cup*. *Taf12L *is a testis-specific TAF whose expression begins in primary spermatocytes and is necessary for normal spermatid differentiation [38]. Both *r-cup *and *f-cup *are expressed at low levels in primary spermatocytes and at higher levels post-meiotically [80]. All three of these genes are expressed in later differentiating male germ cells, as predicted by their inclusion in the gene set. The inclusion of these genes supports the inference that additional genes in this set, including 314 previously unstudied CG genes, function in the testis in somatic cells or differentiating germ cells.

We examined two additional genes from this set using GFP protein trap reporters. The gene *nervana 2 *(*nrv2*) is a Na^+^, K^+^-ATPase β subunit primarily expressed in the nervous system [81]. A GFP protein trap reveals strong expression in the cytoplasm of somatic cyst cells throughout the testis (Figure 3F). Within the germarium, it appears to be expressed in a complex pattern with some weak expression in somatic and germ cells in the anterior portion and then confined expression to somatic cells in the posterior (Figure 3F). We also examined the gene *GlcAT-S*, which is a glycuronytransferase. The protein trap reporter shows expression throughout the apex of the testis with slightly stronger staining within the cytoplasm of hub cells (identified by antibody staining with FasIII) (Figure 3G). In ovaries, the GFP protein trap appears to be expressed non-specifically throughout the germarium (Figure 3G). The inclusion of *Taf12L*, *r-cup*, and *f-cup*, genes known to be expressed late in male germ cell differentiation and the expression of nrv2-GFP and GlcAT-S-GFP in somatic cells within the testis suggest that other genes in this list may be expressed in these cells as well.

By examining expression patterns across these three experiments, we have identified several interesting candidate genes. We wanted to investigate the function of one of these candidate genes in gametogenesis. Based on its high expression pattern in the anterior-most region of the germarium and testes, which include the GSCs and their immediate daughter cells, we choose to test the function of *CG10990*.

### *CG10990 is *the highly conserved ortholog of the mammalian tumor suppressor gene *Programmed cell death 4*

**Figure 4:**
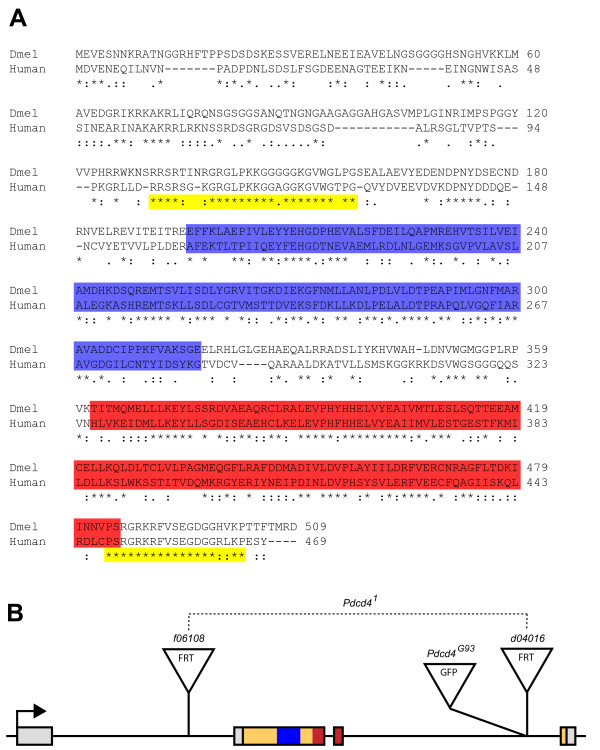
**Sequence similarity and genetic organization of Drosophila *Programmed cell death 4 *(*Pdcd4*, formally called *CG10990*)**. **(A) **Alignment of Pdcd4 protein sequences from *H. sapiens *(human) and *D. melanogaster*. The alignment of identical residues and chemically similar residues are indicated by asterisks and paired dots respectively. The extent of the two MA-3 protein domains are indicated by blue (N-terminal) and red (C-terminal) shading. Yellow shading indicates the extent of highly similar sequences outside the MA-3 domains. **(B) **Organization of the *Pdcd4 *locus. Exons are indicated by rectangles, the transcription start site is indicated by an arrow, open reading frame is shaded yellow, and sequences encoding the two MA-3 domains are shaded blue and red (corresponding to the shading in A). The locations of the transgene insertions are indicated by triangles. The CG10990-GFP protein trap line G00093 is referred to as *Pdcd4^G93^*. The *Pdcd4^1 ^*allele was induced by FRT mediated recombination between the insertions *PBac{WH}CG10990^f06108 ^*and *P{XP}CG10990^d04016 ^*and the region deleted in this mutation is indicated by a dashed line.

Sequence alignments revealed that *CG10990 *shares homology with the mammalian tumor suppressor gene *Programmed cell death 4*. The 509 residue *D. melanogaster *protein sequence shares 37% identical residues and an additional 21% similar residues with the human Pdcd4 (NP055271) protein (Figure 4A). The *D. melanogaster *protein sequence has reciprocal best matches with mammalian Pdcd4 sequences (mouse, rat, and human) indicating that *CG10990 *is the fly ortholog of the mammalian *Pdcd4 *gene. Hereafter we refer to *CG10990 *as *Programmed cell death 4 *(*Pdcd4*).

In mammals, Pdcd4 has been shown to bind to and inhibit the activity of the eukaryotic translation initiation factor eIF4A [82]. Two alpha helical MA-3 domains mediate the binding to eIF4A [83]. The alignment of the fly and human proteins revealed that sequence similarity extends throughout the proteins, including the two MA-3 domains (Figure 4A). The fly and human proteins share 42.6% and 44.4% identical residues over the first and second MA-3 domains respectively (shaded blue and red in Figure 4A). There are also notable stretches of sequence similarity outside the MA-3 domains, including 79% identities over 28 residues in the N-terminal region and 89% identities (100% including similarities) over 19 residues in the C-terminal region (shaded yellow in Figure 4A). The high level of sequence conservation suggests that binding partners may also be conserved between mammals and flies.

### *Pdcd4 *functions to promote the differentiation of female germline stem cells to dividing cystocytes

In order to test the function of *Pdcd4*, we generated a mutant allele using FRT mediated recombination between existing transgene insertions. We induced recombination between *PBac{WH}CG10990^f06108^*, which is inserted in the intron upstream of exon 2, and *P{XP}CG10990^d04016^*, which is inserted in the intron downstream of exon 3 (Figure 4B). We used PCR amplification and DNA sequencing to confirm that the recombinant chromosome bore the expected deletion and hybrid element created as a consequence of the recombination (data not shown). The resulting allele, *Pdcd4^1^*, lacks 95% of the coding sequence including the translation initiation codon and the sequences encoding both MA-3 domains. It is therefore highly likely that the mutation eliminates gene function. We also reasoned that the *CG10990-GFP *protein trap (now referred to as *Pdcd4^G93^*), which results in the fusion of a GFP peptide sequences within the Pdcd4 protein (at the C-terminal end of the second MA-3 domain) may also perturb gene function. We therefore examined both *Pdcd4^1 ^*and *Pdcd4^G93 ^*for possible germline defects.

Both *Pdcd4^1 ^*and *Pdcd4^G93 ^*are hemizygous and homozygous viable with no obvious developmental or morphological defects, indicating that *Pdcd4 *is not essential for organismal viability or development. Males hemizygous for either *Pdcd4^1 ^*or *Pdcd4^G93 ^*were fully fertile and showed no obvious morphological defects in the adult germline (examined by light microscopy). Females of the following genotypes were all found to be fertile and fecund with no obvious morphological defects in the adult germline: *Pdcd4^1^*/*Pdcd4^1^*, *Pdcd4^G93^*/*Pdcd4^G93^*, *Pdcd4^1^*/*Pdcd4^G93^*, *Df(1)ED7217*/*Pdcd4^1^*, *Df(1)ED7217*/*Pdcd4^G93^*. This indicates that *Pdcd4 *function is not essential for spermatogenesis or oogenesis.

Given that in mammals Pdcd4 binds to and inhibits the activity of eIF4A, and the recent finding that Drosophila eIF4A is required for female germline stem cell maintenance [84], we reasoned that *Pdcd4 *may play a role in stem cell maintenance or differentiation. We therefore examined the differentiation of cells in *Pdcd4 *mutants, focusing in particular on female germ cells. In the female germline, the morphology of spectrosomes and fusomes can be used to distinguish GSCs, pre-CBs, and CBs from differentiating germ cells at the 2 cell stage or later (cystocytes). GSCs, pre-CBs, and CBs all contain a single spherical spectrosome, and in dividing cystocytes, the spectrosome undergoes a morphological change to form a branched fusome that occupies the cytoplasmic bridges between cells [85]. We used immunolocalization of anti-Hts, which decorates the spectrosome and fusome, to distinguish GSCs, pre-CBs, and CBs (spherical spectrosomes) from dividing cyst cells (elongated or branched fusome).

**Figure 5:**
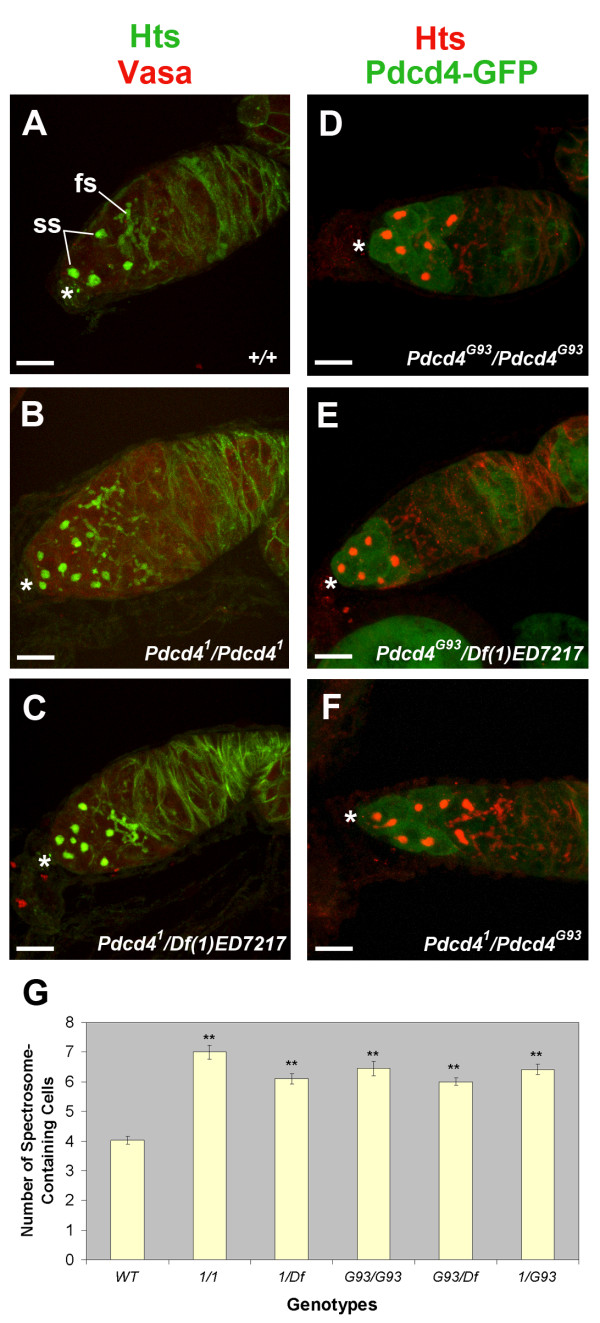
**Loss-of-function *Pdcd4 *mutations result in an increase in the number of cells with a spherical spectrosome**. **(A-F) **Immunofluorescence images showing single projections of confocal Z-stacks of germaria from 5-day-old females. Genotypes are listed in the bottom right corner of each panel. Immunostaining with anti-Hts (green channel in A-C and red channel in D-F) decorated the spectrosomes (ss) and fusomes (fs). Anti-Vasa (red in A-C) decorated germ cells, and the GFP protein trap *Pdcd4^G93 ^*decorated the apical tips of the germaria. Scale bars are 10 μm. **(A) **Representative wild-type germarium showing the typical four cells with spherical spectrosomes. **(B-F) **Representative germaria from *Pdcd4 *mutants showing an increased number of cells with spherical spectrosomes. **(G) **Frequency histogram showing the mean number of cells containing spherical spectrosomes ± standard error for the indicated genotypes. Cells numbers were scored by scanning up and down through confocal Z-stacks. A minimum of 38 germaria from at least 19 females were scored for each genotype. ** indicates a p-value less than or equal to 1 × 10^-16 ^for a comparison between the number of spectrosome-containing cells in the respective mutants vs wild-type.

Examining the distribution of cell types in the germaria revealed that *Pdcd4 *mutants have an increased number of spectrosome-containing cells (Figure 5). Germaria from 5-day old wild-type females contained an average of 4.03 spectrosome-containing cells. However, females containing the *Pdcd4^1 ^*or *Pdcd4^G93 ^*mutant alleles, when homozygous, transheterozygous, or in combination with a deficiency, resulted in an increase in the number of spectrosome-containing cells compared to wild-type. In 5 day old mutants, the average number of cells containing spherical spectrosomes per germarium was between 6.11 and 7.01, and each of the mutant genotypes tested showed a statistically significant increase compared to wild-type. The fact that the mutant phenotype was observed in transheterozygotes, and in individuals bearing each allele in combination with a deficiency, indicates that the phenotype is due to a mutation at *Pdcd4*. The observation that *Pdcd4^1 ^*or *Pdcd4^G93 ^*mutant alleles when homozygous, transheterozygous, or heterozygous with a deficiency resulted in a similar increase in the number of spectrosome-containing cells indicates that at least for this phenotype, both alleles behave as genetic null mutations. The only mutant phenotype observed was an increase of the number cells containing spherical spectrosomes; there were no discernible defects in differentiation or any discernible abnormalities in subsequent germline stages. The morphology of cysts with branched fusomes was indistinguishable from those in wild-type. Finally, in the mutants, the number of spectrosome-containing cells did not increase with age -- similar numbers were observed at 5, 10, and 15 days. These data indicate that wild-type *Pdcd4 *activity promotes the transition from germline stem cell to dividing cystocyte and that the overall transition from GSC to dividing cyst cell is affected in *Pdcd4 *mutants.

The *Pdcd4^G93 ^*protein trap is expressed at a high level in cells at the anterior of the germarium and then decreases in the middle of the germarium. Since *Pdcd4^G93 ^*is a mutant allele that affects the distribution of cell types in the germarium, it is possible that this expression pattern does not reflect the wild-type expression pattern. In *Pdcd4 *mutants bearing this allele (*Pdcd4^G93^*/*Pdcd4^G93^*, *Pdcd4^1^*/*Pdcd4^G93^*, *Df(1)ED7217*/*Pdcd4^G93^*) there was a one-to-one correspondence between the high level of GFP signal and the presence of spherical spectrosomes. Typically, cells containing a spherical spectrosome also showed high GFP signal, and the GFP signal was markedly reduced in cells with dumbbell and branched fusomes. This correlation extended to rare cases where germ cells containing spherical spectrosomes and high levels of GFP were observed posterior to dividing cysts. This indicates that the change in protein expression is associated with the transition to dividing cystocyte and not correlated with proximity to the cap.

### *Pdcd4 *functions in the transition from pre-cystoblasts to cystoblasts

We have shown that *Pdcd4 *mutants have an increased number of cells containing spherical spectrosomes. This could result from an increased number of GSCs, pre-CBs, CBs, or some combination thereof. To determine the identity of the additional spherical spectrosome-containing cells in *Pdcd4 *mutants, we examined the expression of molecular markers that distinguish between GSCs and CBs, specifically pMad and Bam.

**Figure 6:**
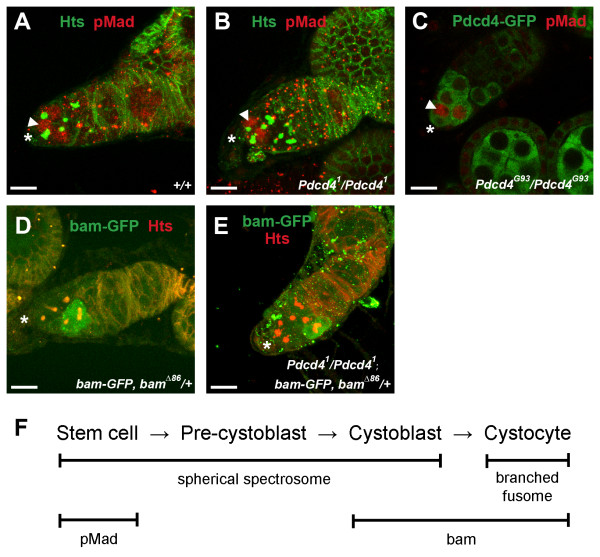
**Loss-of-function *Pdcd4 *mutations result in an increase in the number of pre-cystoblasts**. **(A-C) **Immunofluorescence images showing germaria from 5-day-old females of the indicated genotypes. Immunostaining with pMad (red channel) was used as a marker for GSCs. Anti-Hts (green in A&B) decorated spectrosomes and fusomes and Pdcd4-GFP (the GFP protein trap *Pdcd4^G93^*, green channel in C) reported the expression of Pdcd4. Arrowheads indicate examples of high nuclear pMad expression. **(D-E) **Immunofluorescence images showing single projections of confocal Z-stacks of germaria from 5-day-old females of the indicated genotypes. Immunostaining for bam-GFP (green channel) was used as a marker for CBs, and immunostaining with anti-Hts (red channel) decorated spectrosomes and fusomes. Asterisks indicate the locations of apical tips of the germaria. Scale bars are 10 μm. **(F) **A schematic depicting the relationship between spectrosome/fusome morphology, pMad and Bam expression, and cell identity within the germarium. Pre-cystoblasts are defined as cells containing spherical spectrosomes that do not express pMad or Bam.

Phosphorylated Mad protein (pMad) is part of the signal transduction cascade turned on within GSCs in response to *dpp *signaling from the GSC niche and is thus a marker for GSC identity. pMad is expressed at high levels in GSCs and at very weak levels in pre-CBs and CBs [76,86]. To test the possibility that the additional spherical spectrosome-containing cells in *Pdcd4 *mutants are an expanded GSC population, we used immunostaining with pMad and Hts. Wild-type germaria showed high levels of pMad in GSCs (arrowhead, Figure 6A). Homozygous *Pdcd4^1 ^*germaria also showed high levels of pMad only in those cells adjacent to the niche (Figure 6B), indicating that these ovaries contain a normal population of GSCs. We also looked at homozygous *Pdcd4^G93 ^*germaria immunostained with Pdcd4-GFP and pMad (Figure 6C). Spherical spectrosomes are not highlighted here but as we noted earlier, in the anterior portion of the germarium, there is a one-to-one correspondence between high levels of Pdcd4-GFP and the presence of spherical spectrosomes (Figure 5D, E, and 5F). Only the cells at the anterior-most of the germarium contain high levels of nuclear pMad (Figure 6C). Noting that these cells will also contain spherical spectrosomes, we can conclude that they are GSCs and are present in similar numbers to wild-type. Considering that the other Pdcd4-GFP positive cells at the anterior portion of the germarium contain spherical spectrosomes but not high levels of pMad, we can conclude that they are not GSCs. Antibody staining using pMad as a marker for GSCs clearly demonstrates that the increase in cells containing spherical spectrosomes in *Pdcd4^1 ^*and *Pdcd4^G93 ^*germaria is not due to an expanded population of GSCs.

In females, *bam *is the key intrinsic regulator of differentiation and cystoblasts require Bam for differentiation [5,87]. The *bam-GFP *transgene is a transcriptional reporter that is not expressed at a detectable level in GSCs and pre-CBs but is expressed at high levels in CBs and dividing cysts [88,89]. To test the possibility that the additional spherical spectrosome-containing cells in *Pdcd4 *mutants are an expanded CB population, we used the *bam-GFP *transcriptional reporter to identify CBs. In a wild-type background, the *bam-GFP *reporter construct was expressed at high levels in cystoblasts and early dividing cystocytes (Figure 6D). Most spherical spectrosome-containing cells removed from the niche showed high bam-GFP expression, consistent with CB identity. However, a few spherical spectrosome-containing cells removed from the niche showed no detectible bam-GFP expression, consistent with pre-cystoblast identity (Figure 6D). In a *Pdcd4^1 ^*homozygous background, the majority of the spherical spectrosome-containing cells removed from the niche did not show detectible *bam-GFP *reporter expression (Figure 6E). This suggests that the expanded population of spherical spectrosome-containing cells in *Pdcd4 *mutants are not CBs. Taken together, the marker expression in these cells - no detectible pMad expression and no detectible bam-GFP expression - indicates that they are pre-cystoblasts (Figure 6F).

### *Pdcd4 *interacts genetically with *eIF4A *and *bag of marbles*

We have shown that *Pdcd4 *is required for the efficient differentiation of GSC daughters. In the female germline, *bam *is a key intrinsic regulator of differentiation. In the absence of *bam *activity, GSC daughters fail to differentiate [87], and ectopic expression of *bam *in GSCs is sufficient to induce differentiation [5]. Additionally, in the female germline, eIF4A directly inhibits Bam function and is required for GSC self-renewal [84]. The fact that mammalian Pdcd4 can bind to and inhibit the activity of eIF4A [82,83] raised the possibility that Pdcd4's function in differentiation may be mediated through eIF4A and/or Bam. If this were the case, we may expect a genetic interaction between *Pdcd4 *and/or *eIF4A *and/or *bam*.

**Figure 7:**
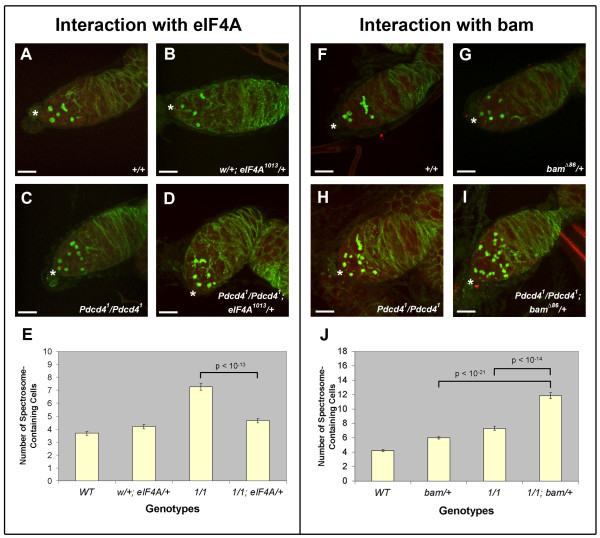
***Pdcd4 *interacts genetically with *eIF4A *and *bam***. **(A-D, F-I) **Immunofluorescence images showing single projections of confocal Z-stacks of germaria from 5-day-old females. Genotypes are listed in the bottom right corner of each panel. Hts is labeled in green and Vasa is labeled in red. Asterisks indicate the locations of apical tips of the germaria. Scale bars are 10 μm. **(E, J) **Frequency histograms showing the mean number of cells containing spherical spectrosomes ± standard error for the indicated genotypes. (E) The p-value shown corresponds to the comparison of the number of spectrosome-containing cells in *Pdcd4^1^/Pdcd4^1^*compared to *Pdcd4^1^/Pdcd4^1^; eIF4A^1013^/+*. A minimum of 34 germaria from at least 17 females were scored for each genotype. (J) The p-values shown correspond to the comparison of the number of spectrosome-containing cells in *Pdcd4^1^/Pdcd4^1^; bam^Δ86^/+ *compared to the control genotypes *bam^Δ86^/+ *and *Pdcd4^1^/Pdcd4^1^*. A minimum of 37 germaria from at least 18 females were scored for each genotype.

We tested whether *Pdcd4 *interacts genetically with *eIF4A *or *bam*. In an otherwise wild-type background, *eIF4A^1013 ^*is recessive and heterozygotes show no significant effect on the number of spherical spectrosome-containing cells (average of 3.78 in wild-type germaria compared to 4.09 in *eIF4A^1013 ^*heterozygotes, Figure 7). Homozygous *Pdcd4^1 ^*germaria contained an average of 7.26 spectrosome-containing cells, and this significantly decreased to 4.65 in *Pdcd4^1^/Pdcd4^1^; eIF4A^1013^/+ *germaria (p-value 2.9 × 10^-13^). Thus, reducing the genetic activity of *eIF4A *acts to suppress the *Pdcd4 *mutant phenotype. In an otherwise wild-type background, *bam *is haploinsufficient with respect to regulating female GSC differentiation, and heterozygosity for a null *bam *mutation results in a increase in the number of CBs [84]. Wild-type germaria contained an average of 4.32 spectrosome-containing cells, which was increased to 7.34 in *Pdcd4^1 ^*homozygotes and 6.01 in *bam^Δ86 ^*heterozygotes (Figure 7). In *Pdcd4^1^/Pdcd4^1^; bam^Δ86^/+ *germaria there were an average of 11.97 spectrosome-containing cells. This was a significant increase compared to the individual mutants (p-value 4.77 × 10^-14 ^compared to *Pdcd4^1 ^*homozygotes and p-value 6.79 × 10^-21 ^compared to *bam^Δ86 ^*heterozygotes). In addition, the range of the phenotype observed in *Pdcd4^1^/Pdcd4^1^; bam^Δ86^/+ *germaria was well beyond anything seen in the individual mutants. For instance, 29% of the double mutant germaria had ≥ 14 spectrosome-containing cells (max: 19), which is well beyond the maximum observed in the individual mutants (*Pdcd4^1 ^*max: 11, *bam^Δ86 ^*max: 8). Thus, *Pdcd4 *and *bam *have a synergistic genetic interaction. The fact that *Pdcd4 *interacts genetically with both *eIF4A *and *bam *strongly suggests that *Pdcd4 *activity impinges on the *bam *and *eIF4A *genetic pathway that regulates the differentiation of female GSCs. Moreover the polarities of the interactions suggest that wild-type Pdcd4 acts genetically in opposition to wild-type eIF4A activity and augments wild-type Bam activity.

## Discussion

Here we report expression profiling of a small population of cells that did not involve genetic manipulations and only minimal physical manipulation of the cells. This identified 2,810 genes with statistically significant sex-biased expression; 1,351 preferentially expressed in the germarium and 1,459 preferentially expressed in the apex of the testis. The inclusion of genes with known sex-specific or sex-biased functions and the validation of a small subset of previously uncharacterized genes provide independent support for these data, and the previously unstudied CG genes are candidate genes for future studies of early gametogenesis. Restricting the analysis of sex-biased gene expression to regions of the gonads containing the earliest stages of adult gametogenesis allowed us to identify genes with sex-biased expression in those specific cells but not in whole gonads. For instance, approximately 31% of the genes identified as sex-biased in this study (24.6% of germarium-biased genes and 37% of apex of the testis-biased genes) do not show sex-biased gene expression in previously published microarray data from whole gonads dissected from adults [18]. These genes have sex-biased expression early in gametogenesis, which is not evident when whole gonads are studied, and represent new candidates for sex-specific or sex-biased function in gonadal somatic cells, germline stem cells, or early stages of gametogenesis.

Combining our experimental data with two previously published data sets allowed us to infer expression patterns in subpopulations of cells. In particular, we focused on three sets of genes: genes expressed in wild-type GSCs and/or daughters cells in both female and male germline stem cell niches, genes expressed preferentially in the germarium but not GSCs, and genes expressed preferentially in the apex of the testis but not in male GSCs, gonialblasts, or somatic stem cells. To our knowledge, this is the first analysis that infers genome-wide gene expression patterns in these cell populations. Examining the cellular expression of two candidates from each class revealed expression patterns consistent with their categorization from the microarray data. Additionally, these analyses identified genes previously known to function in the respective cell types. For instance, the set of genes expressed preferentially in female somatic cells and/or differentiating germ cells contained genes representing several signaling pathways, including the Hedgehog pathway known to be involved in signaling from the terminal filament and cap cells. Conversely, these gene sets included many genes not previously known to function in these cell types. These are candidates for future functional studies. We demonstrated that one of these genes, *Pdcd4*, does have a function in the differentiation of female GSC daughter cells but is not essential for germ cell viability or fertility. It should be noted that such genes may be difficult to detect in mutant screens and that analyses of expression provide a means for their identification.

### The function of *Pdcd4 *in the female germline

The mutant phenotype of loss-of-function *Pdcd4 *was an increase in the number of cells containing a spherical spectrosome, but not an absolute block in differentiation. First, this suggests that *Pdcd4 *is not required to promote the self-renewal of GSCs. Second, it indicates that *Pdcd4 *is not absolutely required for the differentiation of GSC daughter cells. The fact that the number of supernumerary cells did not increase with age - at least between 5 -15 days post eclosion - suggests that the defect is not due to over proliferation but rather a decrease in the efficiency with which GSC daughter cells transition to dividing cyst cells. Our results using pMad as a marker for GSCs and *bam-GFP *as a marker for differentiated CBs indicate that the supernumerary cells with spherical spectrosomes are pre-cystoblasts. Ohlstein and McKearin proposed that pre-cystoblasts must accumulate sufficient cystoplasmic Bam to complete differentiation into CBs [5]. Our demonstration of an enhancing genetic interaction between *Pdcd4 *and *bam *suggests that in *Pdcd4 *mutants, Bam-dependent differentiation of GSC daughter cells may be delayed, leading to an accumulation of pre-cystoblasts.

### A model for the molecular function of *Pdcd4 *in the female germline

Here, we demonstrated an enhancing genetic interaction between *Pdcd4 *and *bam *and an inhibitory genetic interaction between *Pdcd4 *and *eIF4A*. This data, coupled with the known inhibitory protein interaction between mammalian Pdcd4 and eIF4A and the recent finding that eIF4A directly inhibits Bam function within the Drosophila ovary [84], suggests a model for the function of *Pdcd4 *in the transition of GSC daughter cells to dividing cysts. We propose that in GSC daughter cells, Pdcd4 inhibits the activity of eIF4A, thereby relieving eIF4A's inhibition of Bam. In our *Pdcd4 *mutants, we suggest that Pdcd4 is unable to inhibit eIF4A within differentiating GSC daughter cells. This allows eIF4A to continue to inhibit Bam, resulting in a population of pre-cystoblasts that are delayed in their accumulation of sufficient, active Bam levels. Once sufficient Bam levels are reached, then CB differentiation occurs normally. Shen and colleagues proposed that in CBs the inhibition of Bam by eIF4A would be overcome by the high levels of Bam and Bgcn. This is consistent with our model and would explain why *Pdcd4 *is not absolutely required for CB differentiation. Further testing of this model will require the examination of a direct protein-protein interaction between Pdcd4 and eIF4A.

### Does *Pdcd4 *have other functions?

We did not observe any effect of *Pdcd4 *mutations on viability, fertility, fecundity, or morphological development, indicating that *Pdcd4 *does not have a non-redundant essential function (at least under standard laboratory conditions). In mammals, Pdcd4 functions as a tumor suppressor [90], raising the possibility that this function may be conserved in flies. It has recently been found that Drosophila can develop age-dependent tumors of the testes, and at the morphological level, the tumor cells resemble GSCs [91]. Additionally, mutations in the tumor suppressor genes *Salvador *or *Scribble *can result in the transformation of multipotent renal nephritic stem cells (stem cells in adult Malpighian tubules) into cancer stem cells [92]. These observations raise the possibility that *Pdcd4 *may also function as a tumor suppressor in flies.

## Conclusions

The results of our genome-wide expression study on female and male tissue including germline stem cells, somatic niche cells, and early differentiating germ cells identified several sets of candidate genes, some expressed in a sex-biased manner and some identified as expressed in specific cell types. Our analysis of one of these candidate genes shows that *Pdcd4 *functions in females to help promote differentiation in GSC daughter cells. The increase in pre-cystoblast number in *Pdcd4 *germaria, as well as the strong genetic interactions between *Pdcd4 *and *bam *and *Pdcd4 *and *eIF4A*, indicates that *Pdcd4 *plays a role in the germline stem cell differentiation pathway, possibly by relieving the inhibition of Bam by eIF4A.

## Authors' contributions

ACC conducted all of the experimental studies, JA conceived and guided the study, and both authors drafted and approved the final manuscript.

## Supplementary Material

Additional file 1 **Gene expression from microarray experiment comparing germarium to apex of the testis**. Table includes FBgn identifier, gene name, M value, female intensity value, male intensity value, p-value, and FDR adjusted p-value for all genes represented on the microarray. Positive M values indicate enrichment in germaria and negative M values indicate enrichment in testis apex. Click here for file

Additional file 2 **Over-represented biological process GO terms from significant germarium-biased and apex of the testis-biased genes**. GO analysis was performed using the GO Fat feature in DAVID. Table includes GO term, count (number of genes in the list of significant genes with a given term), population hits (number of genes on the microarray with a given term), p-value, and FDR-corrected p-value. Click here for file

Additional file 3 **Genes enriched in wild-type GSCs and/or daughters cells in both females and males**. Table includes FBgn identifier, gene name, final rank order, M value of germarium vs. apex of the testis, female intensity value, male intensity value, adjusted p-value, M value of female GSCs (*dpp*-expanded and *bam*-mutant germ cells) vs. Kc cells [9], adjusted p-value, M value of male stem cells and gonialblasts (*Os^+^bgcn^- ^*testes) vs. *bgcn^- ^*testes [14], adjusted p-value. Click here for file

Additional file 4 **Genes enriched in the germarium but not in female GSCs**. Table includes FBgn identifier, gene name, final rank order, M value of germarium vs. apex of the testis, female intensity value, male intensity value, adjusted p-value, M value of female GSCs (*dpp*-expanded and *bam*-mutant germ cells) vs. Kc cells [9], adjusted p-value. Click here for file

Additional file 5 **Genes enriched in the apex of the testis but not in male stem cells and gonialblasts**. Table includes FBgn identifier, gene name, final rank order, M value of germarium vs. apex of the testis, female intensity value, male intensity value, adjusted p-value, M value of male stem cells and gonialblasts (*Os^+^bgcn^- ^*testes) vs. *bgcn^- ^*testes [14], adjusted p-value. Click here for file
